# “Can virtue be taught?”: a content analysis of medical students’ opinions of the professional and ethical challenges to their professional identity formation

**DOI:** 10.1186/s12909-020-02313-z

**Published:** 2020-10-22

**Authors:** Michael Hawking, Jenny Kim, Melody Jih, Chelsea Hu, John D. Yoon

**Affiliations:** 1 Hematology and Oncology, The University of Chicago, Chicago, IL USA; 2 Department of Biological Sciences, The University of Chicago, Chicago, IL USA; 3 Department of Economics, The University of Chicago, Chicago, IL USA; 4 Department of Economics and the Department of Political Science, The University of Chicago, Chicago, IL USA; 5 MacLean Center for Clinical Medical Ethics, Department of Medicine, The University of Chicago, 5841 S Maryland Ave, Chicago, IL 60637 USA

**Keywords:** Clinical ethics, Kaldjian taxonomy, Professionalism, Professional identity formation, Virtue ethics, Wisdom

## Abstract

**Background:**

Efforts have begun to characterize the ethical and professional issues encountered by medical students in their clinical years. By applying previously identified taxonomies to a national sample of medical students, this study seeks to develop generalizable insights that can inform professional identity formation across various clerkships and medical institutions.

**Methods:**

In a national survey of medical students, participants answered an open-ended survey item that asked them to describe a clinical experience involving an ethical or professional issue. We conducted a content analysis with these responses using the Kaldjian taxonomy of ethical and professionalism themes in medical education through an iterative, consensus-building process. Noting the emerging virtues-based approach to ethics and professionalism, we also reexamined the data using a taxonomy of virtues.

**Results:**

The response rate to this survey item was 144 out of 499 eligible respondents (28.9%). All 144 responses were successfully coded under one or more themes in the original taxonomy of ethical and professional issues, resulting in a total of 173 coded responses. Professional duties was the most frequently coded theme (29.2%), followed by Communication (26.4%), Quality of care (18.8%), Student-specific issues of moral distress (16.7%), Decisions regarding treatment (16.0%), and Justice (13.2%). In the virtues taxonomy, 180 total responses were coded from the 144 original responses, and the most frequent virtue coded was Wisdom (23.6%), followed by Respectfulness (20.1%) and Compassion or Empathy (13.9%).

**Conclusions:**

Originally developed from students’ clinical experiences in one institution, the Kaldjian taxonomy appears to serve as a useful analytical framework for categorizing a variety of clinical experiences faced by a national sample of medical students. This study also supports the development of virtue-based programs that focus on cultivating the virtue of *wisdom* in the practice of medicine.

**Supplementary information:**

The online version contains supplementary material available at 10.1186/s12909-020-02313-z.

## Background

Ethics and professionalism education has become a standard part of medical school curricula [1, 2], and it tends to occur in large and small group settings in the pre-clinical years before students start to have direct encounters with ethically and professionally challenging situations. Placing ethics education in the pre-clinical years is therefore limiting, for at least three reasons: it neglects what is widely recognized as the longitudinal nature of ethics formation [2]; it does not provide opportunities for practical reasoning when students most need it as they encounter concrete ethically complex clinical situations [3]; and the clinical clerkships contain powerful experiences conducive to professional identity formation [4–6] in which students learn to navigate ethical or professional issues that arise over the course of learning to care for patients.

Efforts have begun to characterize the ethical and professional issues encountered by medical students in their clinical years [7–9]. These efforts have been helpful in shining light on the unique moral situations that arise in clinical medicine, but have largely been limited to studies within particular medical schools without wider validation. For example, at the University of Iowa Carver School of Medicine, Kaldjian et al. developed a taxonomy that categorized a wide range of ethical and professional challenges that students in their clinical years highlighted during formalized reflections [3]. The Kaldjian taxonomy included 7 major coded themes (Decisions regarding treatment, Communication, Professional duties, Justice, Student-specific issues, Quality of care, and Miscellaneous) which attempted to capture a wide range of experience-based observations from medical students rather than relying on abstract concepts in ethics and professionalism curricula. Given that this was limited to a single institution, we wanted to test whether this same taxonomy would prove useful when analyzing data from a national sample of medical students, thus yielding more generalizable insights from the taxonomy to inform professional identity formation more broadly.

Therefore, in this present investigation, we made use of a 2011 national medical student dataset [10, 11] to test whether the Kaldjian taxonomy could be applied to a variety of ethical or professional issues faced by students across multiple institutions who were completing their clinical years. Moreover, given the emerging virtues-based approach to ethics and professionalism education [12–14], we further attempted to develop a virtues-based taxonomy of the students’ reported ethical or professional issues by re-coding the same data based on whether certain *virtues* (understood as particular desirable traits of character for a physician to have) were *present* or *absent* in the clinical situations that students described. The list of virtues in our taxonomy was derived from a study that examined which ethical values recurred in the medical oaths taken at various medical schools in the United States and Canada [14].

## Methods

### Survey procedure

Participants were recruited to participate in a 2011 national study of the professional development of physicians-in-training. In September 2009, samples were drawn from the American Medical Association Physician Masterfile, which has a near-complete listing of students pursuing M.D. degrees at schools within the U.S. and its territories. To construct our target sample, we selected 960 third-year students from 24 allopathic medical schools in the U.S. using a two-stage sample design. In stage one, we selected schools with probabilities proportional to total enrollment so that the larger schools would have a greater chance of being included in the study. Data for allopathic medical school sampling was obtained from published reports. In stage two, we used simple random sampling to select a fixed number of students (40) from each selected school.

After a relevant literature review in medical ethics/professionalism, survey questions underwent expert review by colleagues, as well as pre-testing by a group of third-year medical students at one University in the U.S. Midwest. Quantitative data collection was conducted in two phases: administration of self-report Questionnaire 1 occurred between January and April 2011 and assessed demographic variables including gender, race/ethnicity, immigration history, specialty intention, social mission score of the medical school, and other information about medical school experiences (Time 1; students were third-year); a re-contact phase administering Questionnaire 2 occurred six to nine months later to those students who responded to Questionnaire 1 (Time 2; third-year students had become fourth-year). Participants were paid $5 for completion of the first questionnaire, $10 for completion of the second questionnaire. Participants in this study were generally third-year medical students (94.8%). Using a combination of postal mailings and email links to the online versions of the questionnaires, we obtained a response rate of 63% for Time 1 (605/960). At Time 2 our sample size decreased to 499 participants.

### Content analysis

In the Time 2 Questionnaire, we asked the following open-ended survey item which had been adapted from a previous study [3]: “*Lastly, please describe a clinical experience you observed that, in your opinion, raised an ethical or professional issue. Then describe how you thought the situation should have been approached.”* Out of a total of 499 respondents, only 144 respondents (28.9%) offered a legible response to this survey item.

We performed content analysis of each of the responses (*n* = 144), which ranged from single-sentence answers to long paragraph responses. Three undergraduate student investigators (JK, MJ, CH) were provided an initial list of codes from the original Kaldjian taxonomy [3] which contained codes for 7 major themes (Decisions regarding treatment, Communication, Professional duties, Justice, Student-specific issues, Quality of care, and Miscellaneous) and sub-codes for 32 sub-themes (listed in Table 1). Using this list, these three student investigators independently coded each survey response and reached consensus on the ethical and professional issues deemed present. Coded text was entered into NVivo 12.1 (QSR International, 2018). One internal medicine fellow investigator (MH) and two faculty investigators (JY and LK), all of whom have been formally trained in clinical ethics or philosophy, then reviewed all the coding results to confirm agreement. Then through an iterative consensus-building process with the student investigators, further adjustments to major codes and sub-codes were finalized. In the minority of cases when coding of responses did not reach unanimous consensus within the team, the final decision was made by the faculty investigator with the most familiarity of the original taxonomy (LK). In this way, we developed a final list of major codes and sub-codes that provided a summary analysis of students’ responses.

**Table 1:** Ethical or Professional Issues Encountered During Clinical Training: Coded Themes/Sub-Themes from a National Survey of U.S. Fourth Year Medical Students (*N* = 144)

Major Coded Themes	Sub-Themes^**a**^	Count N (%)
**Professional Duties**	Disrespectful treatment of patient or family	15 (35.7)
	Extent or fulfillment of fiduciary responsibilities of healthcare provider	7 (16.7)
	Disrespectful remarks to or about colleagues	4 (9.5)
	Lack of respectful collaboration with colleagues	4 (9.5)
	Lack of self-control	4 (9.5)
	Deception, cheating, or misconduct in training	4 (9.5)
	Lack of professionalism (not otherwise defined)	4 (9.5)
	**Subtotal**	**42 (29.2)**
**Communication**	Inadequate communication	19 (50.0)
	Deliberate lies and deception in context of medical care	7 (18.4)
	Delivering bad news	5 (13.2)
	Breaking adult or adolescent patient confidentiality	4 (10.5)
	Disclosing medical errors	3 (7.9)
	*Subtotal*	**38 (26.4)**
**Quality of Care**	Not meeting standard of care	17 (63.0)
	Medical errors	7 (25.9)
	Treatment of pain	3 (11.1)
	*Subtotal*	**27 (18.8)**
**Student-specific Issues of moral distress**	Willingness to ask critical questions or speak up when concerned	7 (29.2)
	Uncertainties about role and scope of responsibility	4 (16.7)
	Role of religious beliefs in medicine	4 (16.7)
	Feedback on performance and etiquette	4 (16.7)
	Struggle over patients’ lifestyle choices	3 (12.5)
	Learning on patients over their objections or without consent	1 (4.2)
	Learning on patients without supervision or adequate skills or training	1 (4.2)
	*Subtotal*	**24 (16.7)**
**Decisions Regarding Treatment**	Problems surrounding surrogate decision-making	7 (30.4)
	Physician disagreement with patients/surrogates over interventions	6 (26.1)
	Problems surrounding informed consent	4 (17.4)
	Decisions related to continuing life-sustaining treatments	3 (13.0)
	Unclear decision-making capacity of patient	2 (8.7)
	Inter-professional disagreement about patient’s best interests	1 (4.3)
	*Subtotal*	**23 (16.0)**
**Justice**	Discriminatory treatment	7 (36.8)
	Inadequate level of health care	7 (36.8)
	Wasteful or excessive level of healthcare	4 (21.1)
	Allocation of resources	1 (5.3)
	*Subtotal*	**19 (13.2)**
**Total**		**173**^**a**^

^a^Total coded counts are greater than *N =* 144 since some themes were coded multiple times across different categories. Percentages in bold are calculated using a denominator of 144. Percentages in normal text are calculated using the respective sub-total of each of the six categories

We then re-examined students’ responses using a virtues taxonomy utilizing a list of virtues most commonly referenced in a study of medical oaths used in North American medical schools [14]. Student investigators (JK, MJ, CH) were provided a list of these 16 virtues (listed in Table 2) and their corresponding Oxford English Dictionary definitions (Additional file 1). Then using similar procedures as noted above, we coded all the responses using the virtues categories through an iterative, consensus-building process. As noted above, when coding of responses did not reach unanimous consensus within the team, the final decision was made by the faculty investigator with the most familiarity of the original list of virtues (LK).

**Table 2:** Ethical or Professional Issues Encountered During Clinical Training: Coded Themes/Sub-Themes and Examples of Responses from a National Survey of U.S. Fourth Year Medical Students (*N* = 144)

Major Coded Themes	Sub-Themes (***and Examples of Students’ Responses***)
**Professional duties**	Disrespectful treatment of patient or family *“A pediatrician I worked with told an obese boy that if he did not lose weight, he would go blind and they would have to amputate his limbs because of DM. This could have been explained more gently...”*
	Extent or fulfillment of fiduciary responsibilities of healthcare provider *“A community hospital was requiring its physicians to only refer to specialists that were within the hospital’s network of employees. Presumably, this strategy was intended to maximize profits at the potential expense of patient care…”*
	Disrespectful remarks to or about colleagues *“A lot of the services at the hospitals where I’ve had rotations have acted unprofessionally with regards to “talking bad” about other services...”*
	Lack of respectful collaboration with colleagues *“I feel that sometimes patient care is compromised when physicians from different specialties disagree as to patients’ treatment plans and allow the arguments to become…"turf battles.”*
	Lack of self-control *“I once saw a surgeon become frustrated with a new computer system and throw a phone across the room…”*
	Deception, cheating, or misconduct in training *“I have seen other medical students lie about their presence at rotation sites in order to achieve honors in that rotation…”*
	Lack of professionalism (not otherwise defined) *“Multiple times, I’ve seen religion infused into a medical situation where it did not belong…”*
**Communication**	Inadequate communication *“Poor communication with the family of a terminal patient. The situation should have been approached with greater sensitivity, time for discussion, and more openness.”*
	Deliberate lies and deception in context of medical care *“OBGYN resident offered a makeshift “fertility” test to a patient just to appease the patient and her husband, despite knowing this test was not efficacious or even [done] correctly…”*
	Delivering bad news *“I’ve observed a couple situations where nobody really wanted to be the one to give the bad news… I felt that the doctors should have been more straightforward with the patients and their families instead of beating around the bush...”*
	Breaking adult or adolescent patient confidentiality *“Patient with CA had prognosis given to him in crowded waiting room, and doctor had to yell because the patient was hard of hearing. Ideally [the] patient should have been taken somewhere private.”*
	Disclosing medical errors *“A patient was inappropriately treated by her PCP who was a provider outside our facility…I felt we should have informed the patient more explicitly that an error was made…”*
**Quality of Care**	Not meeting standard of care *“Resident refused to properly pad patient’s diabetic ulcers because she was going to be anesthetized for the surgical procedure. Except she was only lightly anesthetized and discomfort endangered the procedure and the patient’s safety…”*
	Medical errors *“Taking a patient to the OR without a CT scan - surgery aborted due to unforeseen anatomy. Better foresight needed.”*
	Treatment of pain *“Giving homeless drug-seekers pain meds to get them to leave the hospital, knowing they had no care or follow-up.”*
**Student-specific issues of moral distress**	Willingness to ask critical questions or speak up when concerned *“Recently witnessed a neurologist spray water into a coma patient’s mouth as she said, “he was thirsty,” and then laughed. Unfortunately, [because] she’s my instructor/grader I felt helpless….”*
	Uncertainties about role and scope of responsibility *“Resident asked me to write notes under their computer log-in. I told her I did not feel comfortable doing so and would prefer to write notes under my own credentials.”*
	Role of religious beliefs in medicine *“I worked with a pediatrician who was a strong Christian who did not support birth control or abortions. His beliefs were significantly impacting his care and recommendations to patients…”*
	Feedback on performance and etiquette *“I watched an intern being put down and mistreated by an attending in front of a patient. It shouldn’t have happened or the attending should have apologized to everyone involved.”*
	Struggle over patients’ lifestyle choices *“Caring for a liver failure “frequent flyer”, the team felt little compassion for the patient. The [patient] had made clearly poor life choices, however, I don’t believe this should have changed the amount/quality/completeness of his care…”*
	Learning on patients over their objections or without consent *“A patient anesthetized for a surgery whom we then were asked to perform a pelvic exam to which she had not been asked or consulted to. I followed directions [because] I was unsure but wish I had not.”*
	Learning on patients without supervision or adequate skills or training *“I have had a patient in the SICU [whose] prognosis is poor and the intern needed to place an arterial line. The attempts were unsuccessful and I felt there was more attempts made than should have been because she was sedated and he needed to complete the task.”*
**Decisions regarding treatment**	Problems surrounding surrogate decision-making *“There was a patient in my IM rotation whose POA seemed uninterested in his healthcare and never visited, was difficult during phone conversations. I felt as though we should have consulted ethics to see if we were providing the care that the patient really desired/wanted…”*
	Physician disagreement with patients/surrogates over interventions *“Patient with poor life expectancy…[and] wife did not want hospice or to withdraw care that likely was not helping. Palliative care consulted, but wife continued to want aggressive management…[T]eam eventually grew frustrated and antagonized [patient]‘s wife.”*
	Problems surrounding informed consent *“One individual, a patient, was about to undergo a procedure. He did not speak English. When I spoke to him in his native language (in which I am fluent), it became clear that no one had properly informed him of what was going to happen or what he had consented to…”*
	Decisions related to continuing life-sustaining treatments *“A patient has been on full life support for over 1 year now, because the spouse refuses to take them off…The family should be talked to again and perhaps get higher authority involved to help overrule the decision.”*
	Unclear decision-making capacity of patient *“A man in the hospital was dying, and his next of kin was desperately asking the patient if it was ok for him to be comfort measures only. The patient was past the point when he could talk, but eventually weakly nodded “yes.” Ideally, this [evaluation] of life conversation would have started when the man was well…”*
	Inter-professional disagreement about patient’s best interests *“Terminally ill 14 yo girl with Acute Lymphocytic Leukemia complaining of severe pain and neuropathic symptoms. Neurology consulted to evaluate and recommended an EMG…I found [the study] to be inappropriate for the situation…”*
**Justice**	Discriminatory treatment *“Many physicians treat patients at the county hospital differently than other populations or talk derisively of them behind their backs...”*
	Inadequate level of health care *“While working on the substance abuse unit a ‘no smoking’ policy was enforced. Nicotine patches were not provided for the residents and a few ended up leaving early because they could not handle the stress of the addiction and not smoking. I think that Wellbutrin, Chantrix, or nicotine patches or gum should have been provided…”*
	Wasteful or excessive level of healthcare *“[E]xtra lists ordered to “rule-out” conditions for state-funded hospital stays* versus *more cautious ordering for private pay.”*
	Allocation of resources *“…[O]n a few instances, family members have prolonged the suffering of terminally ill patients…I think more physicians should be more assertive and/or aggressive in managing these patients with their well-being in mind, as well as conservation of resources*

We approached data analysis with a post-positivist lens, by acknowledging the potential effects of our personal biases. Two authors (JK, CH) had previous training in qualitative data analysis, and together with the faculty investigator (JY) trained the rest of the student team to use these methods rigorously. Another investigator (LK), an experienced clinician-ethicist who was also well-versed in qualitative data analysis, helped guide theoretical discussions along with the other authors (JY and MH). Together, our combined expertise allowed for a careful examination of the data while our staged, iterative approach helped protect against potential biases.

This study was approved by the Institutional Review Board in January 2011. The illustrative examples in the supplementary table (see Additional file 1, available online only) have been modified in non-essential respects to remove identifiable information.

## Results

Table 1 shows how students’ ethical or professional issues encountered during clinical training were categorized using Kaldjian’s taxonomy of ethical and professional issues, leading to 6 major coded themes: 1) Professional duties 2) Communication 3) Quality of care 4) Student-specific issues of moral distress 5) Decisions regarding treatment and 6) Justice. Table 1 also shows the further coding of responses according to the Kaldjian taxonomy’s 32 sub-themes, for which example responses are provided. From 144 students, we were able to code 173 separate responses since some responses were coded across multiple categories. In contrast to the original taxonomy, in our slightly modified version we did not include a “Miscellaneous” category (which accounted for 9.8% of responses in the original taxonomy) and we further specified the original “Student-specific issues” category as “Student-specific issues of moral distress.” While all responses were able to be categorized using these 6 major themes, we noted differences in the frequencies of many sub-themes between the two versions of the taxonomy. Among the 6 major themes common to both the modified and original versions, we observed the following comparative frequencies: Professional duties (29.2% modified vs. 18.4% original, *p* = 0.007), Communication (26.4% modified vs. 21.4% original, *p =* ns), Quality of care (18.8% modified vs. 3.8% original, *p* < 0.001), Student-specific issues of moral distress (16.7% modified vs. 5.4% original, *p <* 0.001), Decisions regarding treatment (16.0% modified vs. 31.4% original, *p* = 0.004), and Justice (13.2% modified vs. 9.8% original, *p =* ns). Examples of students’ responses for the various themes and sub-themes are noted in Table 2 with actual quotations from the student respondents. Table 3 shows the frequencies of responses categorized by the virtues taxonomy, as well as examples of student responses for each virtue category. From 144 students, we were able to code 180 separate responses since some responses were coded across multiple categories using the virtues taxonomy. The most frequently coded virtues in our data included Wisdom (*N* = 34, 23.6%), Respectfulness (*N* = 29, 20.1%), and Compassion or Empathy (*N* = 20, 13.9%). We also found that some virtues (whether demonstrated by their presence or absence) were infrequently coded (Self-reflection, Courage, Altruism) or not coded at all (Forgiving, Gratitude).

**Table 3:** Virtues Relevant in the Professional or Ethical Issues Encountered During Clinical Training: Coded Themes from a National Survey of U.S. Fourth Year Medical Students (*N =* 144)

Major Coded Themes	Examples of Students’ Responses	Count N (%)
**Wisdom**	*“Dealing with a patient who had a history of drug-seeking behavior … I think it is important to initially take them at their word, but remain vigilant so as not to be taken advantage of*.”	34 (23.6)
**Respectfulness**	*“[O]bserved a physician being abrasive towards a [patient] to persuade them not to have a procedure.”*	29 (20.1)
**Compassion or empathy**	*“Residents speaking to patients rudely, increased volume. I thought the resident should be more compassionate.”*	20 (13.9)
**Uprightness**	*“[T]he attending had us sign in under her name. We would write her notes to place her orders…”*	16 (11.1)
**Honesty**	*“A senior resident blatantly lied on rounds about a patient…”*	15 (10.4)
**Conscience**	*“I have seen many residents cut corners which compromised patient care. Many of these situations were when a resident didn’t know what to do and rather than ask or read, made an un-informed poor decision.”*	14 (9.7)
**Honor or integrity**	*“…[My OB/GYN] attending was very involved in the business aspect of his practice…Despite new recommendations on PAP smears, he continued to do them on women who probably did not need them…if patients asked why they needed them, he would avoid their questions…”*	12 (8.3)
**Regard as human being**	*“Hospital’s treatment of gunshot wound of a gang member* vs *an officer: huge, startling discrepancy.”*	11 (7.6)
**Patience**	*“I worked with one resident who was rude and completely ignored a patient’s feelings. He started his H&P firing off questions without waiting for responses and cutting her off…”*	8 (5.6)
**Humility**	*“I once saw a doctor yelling at a critical care nurse because she was trying to clarify an order for a patient…”*	6 (4.2)
**Charity**	*“The family wanted the neurosurgery team to speak with them…before withdrawing life support. The neurosurgery team refused to speak with family stating “there is nothing more they are able to relay to the family or do for the patient.”*	6 (4.2)
**Self-reflection**	*“We all have biases, and I think it’s important that we engage in discussion on what they are. This is especially important for physicians to be aware of…”*	4 (2.8)
**Courage**	*“The issue of a medical student not being able to stick up for herself/himself because they are worried about how they will be graded is an issue…”*	3 (2.1)
**Altruism**	*“Turning away a [patient] based on ability to pay…”*	2 (1.4)
**Forgiving**	*[no codes identified]*	0
**Gratitude**	*[no codes identified]*	0
**Total**		**180**^**a**^

^a^ Total may greater than *N =* 144 since some themes may have been coded multiple times across different categories. Percentages are calculated using a denominator of 144

## Discussion

Our study found that the Kaldjian taxonomy of ethical and professional issues was able to categorize the wide range of themes and sub-themes found in the ethical and professional issues provided by the students we studied. This is notable since our respondents differed from those in the Kaldjian study by being drawn from a national sample rather than a single institution. In addition, the Kaldjian sample was taken from responses written during (and drawn from) clerkships in internal medicine and pediatrics, while our students’ responses were not clerkship-specific. Some of the variance in theme frequencies between the two studies could represent differences in culture and exposure across institutions or possibly across clerkships. Nonetheless, having tested this taxonomy in a national sample of medical students, we can be more confident that this taxonomy reliably captures the range of ethical and professional issues that medical students experience in medical school.

Mindful of the broader rationalist and intuitionist divide in approaches to ethics education [12], we also used a virtue taxonomy to code student responses. As described above, this taxonomy was derived from medical school oaths across the United States and Canada. We take it that the particular medical virtues included in the taxonomy, recognized in the oaths as traits or dispositions worth cultivating, are good for physicians to have. Taken together, these traits enable a physician to account for the morally relevant circumstances in a given clinical situation and respond in an appropriate and just manner [15]. The usefulness of this taxonomy contributes helpful information to ongoing discussion about the merits of adopting a virtue-based moral framework in medical ethics education [13, 16, 17].

In particular, it is notable that *wisdom* was the virtue most often deemed most relevant across a diverse set of students’ clinical experiences. This finding supports the idea of developing premedical undergraduate and medical school curricula and “communities of practice” [18], in which students and teachers can reason together and learn how to become *good physicians* through role modeling and continual refinement. As Aristotle noted in the *Nicomachean Ethics* [19], moral decision making often involves so much complexity that no set of algorithmic decision trees or list of principles could be comprehensive enough to generate a response—and so it is in medicine. Algorithms or lists of moral rules can be useful in medicine in a limited sense, but clinical decision making, with all of its medical, social, and ethical complexity, eludes reductionist approaches.

In an effort to promote virtue in general, and wisdom in particular, among future physicians, we would endorse a model of professional identity formation that is based around mentorship in intentional learning communities where the “lived experiences of mentors and learners” are brought into conversation in a didactic manner [20]. Such educational models tend to emphasize the beneficial use of narrative, the creation of an engaged community of learners, and intentional reflective processes in a longitudinal curriculum that fosters an “apprenticeship” model of clinical education [21, 22]. Within such a learning community, the Kaldjian taxonomy provides a possible curricular roadmap for professional identity formation in which students would be challenged to think through specific kinds of case scenarios that reflect the major content themes of the taxonomy. Moreover, students would be inspired to cultivate the virtue of wisdom within a community of role model physicians who embody such wisdom in the way in which they practice medicine. By reflecting together over the relevant case scenarios that are most frequently encountered in medical students’ clinical experiences, this taxonomy thus serves as a possible framework for reflecting together and teaching wisdom, with the goal of helping future physicians learn how to reason through real clinical situations that are medically, socially, and ethically complex.

There are several limitations to our study. First, the students’ responses are subject to recall bias, since the data are necessarily retrospective. Second, the original Kaldjian taxonomy was developed from students’ reflections that were much longer and detailed in content than the brief responses provided by students in our national survey (i.e. responses were limited to a few sentences or a paragraph). Therefore, our coding process may not have had the same level of coding specificity found in the original Kaldjian study in which the students were expected to provide much longer responses in the context of a mandatory class assignment (as opposed to a voluntary mail/online survey). However, we tried to minimize bias through the use of multiple investigators examining the same data. Additionally, the survey from which this analysis was conducted was collected in 2011, thus limiting the applicability of our findings to the present landscape of medical education in the United States. However, we feel that the issues of ethics, professionalism, and virtues brought up by the student participants remain relevant, as recent studies have continued to find and elaborate on similar themes in medical students’ educational experiences [23–26]. Lastly, though we achieved a good overall response rate for the national survey, the survey item used in the current study had a completion rate of 28.9% (144 out of 499). Non-respondents may have differed from respondents in ways that bias our results.

## Conclusions

Originally developed from students’ clinical experiences in one institution, the Kaldjian taxonomy appears to serve as a useful analytical framework for categorizing a wide variety of ethical and professional issues encountered in the clinical experiences of a national sample of medical students. This study also supports the development of virtue-based programs that focus on cultivating the virtue of *wisdom* in the practice of medicine.

## Supplementary information

Additional file 1. Oxford English Dictionary Definitions of Virtues Used During Thematic Coding. (DOCX 25.8 kb)

## Data Availability

Data is made available at request.
