# Supplementary material for: “Can virtue be taught?”: a content analysis of medical students’ opinions of the professional and ethical challenges to their professional identity formation
Source: BMC Med Educ. 2020 Oct 22;20:380. doi: 10.1186/s12909-020-02313-z (PMC7584068; doi:10.1186/s12909-020-02313-z)
Supplement: Supplementary file 1 — Oxford English Dictionary Definitions of Virtues Used During Thematic Coding. (DOCX 25.8 kb) [file 12909_2020_2313_MOESM1_ESM.docx]

**Additional file 1**

**Oxford English Dictionary Definitions of Virtues Used During Thematic Coding**

**Wisdom:** Capacity of judging rightly in matters relating to life and conduct; soundness of judgement in the choice of means and ends; sometimes, less strictly, sound sense, esp. in practical affairs: opposed to folly.

**Respectfulness:** (respectful) Mindful, heedful, careful (of something). Of a person: full of or exhibiting respect; deferential. Of a gesture, attitude, etc.: indicative of or marked by respect.

**Compassion/empathy:** The feeling or emotion, when a person is moved by the suffering or distress of another, and by the desire to relieve it; pity that inclines one to spare or to succor

**Uprightness:** The state or condition of being sincere, honest, or just; equity or justness in respect of principle or practice; upright quality or conduct; moral integrity or rectitude.

**Honesty:** (honest) Of an action, feeling, etc.: done with or expressive of truthfulness, fairness, or integrity of character or intention; free from deceit; genuine, sincere. Also: done with good intentions even if unsuccessful or misguided.

**Conscience:** The internal acknowledgement or recognition of the moral quality of one's motives and actions; the sense of right and wrong as regards things for which one is responsible; the faculty or principle which judges the moral quality of one's actions or motives. Now also in weakened sense: one's awareness of what is advisable or acceptable for one to do.

**Honor/integrity:** (Honor) Great respect, esteem, or reverence received, gained, or enjoyed by a person or thing; glory, renown, fame; reputation, good name. (Integrity) Soundness of moral principle; the character of uncorrupted virtue, esp. in relation to truth and fair dealing; uprightness, honesty, sincerity.

**Regard as human being:** (Regard) Detailed consideration of a problem or question; notice taken of a person or thing; close attention.

**Patience:** The calm, uncomplaining endurance of pain, affliction, inconvenience, etc.; the capacity for such endurance.

**Humility:** The quality of being humble or having a lowly opinion of oneself; meekness, lowliness, humbleness: the opposite of pride or haughtiness.

**Charity:** Love, kindness, affection, natural affection, now esp. With some notion of generous or spontaneous goodness.

**Self-reflection:** Reflection on or serious thought about one’s character, actions, motives, etc.; introspection.

**Courage:** That quality of mind which shows itself in facing danger without fear or shrinking; bravery, boldness, valor.

**Altruism:** Disinterested or selfless concern for the well-being of others, esp. as a principle of action.

**Forgiving:** Inclined to forgive. (Forgive) To give up resentment against, pardon (an offender); to abandon one’s claim against (a debtor).

**Gratitude:** The quality or condition of being grateful; a warm sense of appreciation of kindness received, involving a feeling of goodwill towards the benefactor and a desire to do something in return; gratefulness.
